# Recommendation of RILEM TC 281-CCC: RILEM CPC-18R1—guideline for measuring the carbonation depth of hardened concrete using a pH indicator solution

**DOI:** 10.1617/s11527-026-02966-0

**Published:** 2026-03-04

**Authors:** Susan A. Bernal, Ueli M. Angst, John L. Provis, Charlotte Thiel, Gregor J. G. Gluth, Yury Villagran-Zaccardi, Nele De Belie

**Affiliations:** 1 Department of Architecture and Civil Engineering, University of Bath, Claverton Down, Bath, BA2 7AY UK; 2 Durability of Engineering Materials, Institute for Building Materials, ETH Zürich, 8093 Zurich, Switzerland; 3 PSI Center for Nuclear Engineering and Sciences, Paul Scherrer Institut, Forschungsstrasse 111, 5232 Villigen PSI, Switzerland; 4 Construction Materials, OTH Regensburg, Regensburg, Germany; 5 Division 7.4 Technology of Construction Materials, Bundesanstalt für Materialforschung und -Prüfung (BAM), Unter den Eichen 87, 12205 Berlin, Germany; 6 Materials and Chemistry Unit, Flemish Institute for Technological Research (VITO), 2400 Mol, Belgium; 7 Magnel-Vandepitte Laboratory for Structural Engineering and Building Materials, Ghent University, Technologiepark Zwijnaarde 60, Campus Ardoyen, 9052 Ghent, Belgium

**Keywords:** Carbonation, Carbonation depth, Carbonation front, pH indicator, RILEM CPC-18, Test method

## Abstract

This recommendation provides a procedure for determining the carbonation depth on the surface of concrete by applying a pH indicator. This includes definitions of carbonation, carbonation depth and carbonation front, as well as descriptions of the different pH indicator solutions that can be used. Recommendations for testing laboratory-prepared specimens and those obtained from concrete structures are also given. This involves guidelines for sample preparation and/or extraction, CO_2_ exposure duration, carbonation depth determination and reporting of results. A section on data interpretation is also provided, as carbonation results are used for determining durability of concrete, as well as a criterion for materials selection or for carbon uptake calculations. The new Recommendation CPC-18R1 is intended to supersede the former RILEM recommendation CPC-18, particularly when prescribed as the preferred method for evaluating and reporting carbonation depths.

## Introduction

The RILEM Concrete Permanent Committee (CPC) [1] created in 1988 the CPC-18 recommendation ‘*Measurement of hardened concrete carbonation depth’* [2] which served as a foundation for the development of later European standards to determine carbonation resistance of concrete. Since its creation, there have been significant developments in standards and guidelines for determining carbonation resistance of concrete, which are described and analysed in detail in [3]. The RILEM CPC-18 recommendation is cited in existing standards including BSI 1881–210 [4] and CUR-Aanbeveling 48:2010 [5], and it is extensively adopted in practice in regions where no standards for carbonation assessment exist. This motivated the revision of this recommendation by an Editorial Committee of the RILEM TC 281-CCC ‘Carbonation of concrete with supplementary cementitious materials’, to ensure that this recommendation is in line with the current state of the art, and integrates the advances in understanding of carbonation of concrete since the original CPC-18 was published. This recommendation also aims to assist in the interpretation of results obtained using a pH indicator solution to determine the carbonation depth of concrete.

## AIM and scope

This Recommendation presents a procedure for determining the depth of the carbonated layer on the surface of hardened concrete by application of an indicator. This method can be carried out using specimens made in the laboratory, and on specimens obtained from complete structures or structural members (referred to as site specimens). It can also be used for testing specimens that have been exposed to natural carbonation on-site. It is also the simplest and fastest method to draw conclusions about the potential progress of carbonation.

The indicator method can detect changes in pH, if the pH change is sufficiently pronounced to lead to a change in colour of the selected pH indicator solution. If applied on a concrete surface, the location where the colour change occurs may be used to determine the actual carbonation depth.

Knowledge of pH changes in concrete, is typically associated with the depth of carbonation, and can be of interest in different contexts. For instance, the pH is a critical factor influencing corrosion of steel reinforcement [6], and so there is a long tradition in concrete condition assessment practices to compare carbonation depths with the cover layer depth. Moreover, in the context of carbon dioxide sequestration by concrete, changes in pH provide important information regarding the potential extent and depth to which CO_2_-storing reactions might have occurred. However, it must be noted that there is not a well-established correlation between pH changes and the quantity of carbonates formed in cementitious materials upon carbonation. If accurate quantification of carbonates is required, extraction of paste samples at different cover depths is recommended instead. Paste samples can then be analysed by different analytical techniques (e.g. thermogravimetry, quantitative X-ray diffraction, or others) according to recommendations described in [7].

Tests conducted on laboratory specimens are generally used to enable comparison of the rates of carbonation in specimens of various concrete mixes, exposed under defined (ambient or elevated CO_2_ concentrations), and often comparable (e.g. temperature, relative humidity), conditions. The results of such tests enable ranking of concretes in terms of carbonation resistance, and can assist in materials selection. Tests carried out on-site, or using on-site specimens, also referred to as natural carbonation, are necessary to gain information regarding the carbonation extent in a concrete structure or structural member at a certain moment in time, and under specific microclimates or in-service exposure conditions.

This recommendation aims to enhance the comparability and accuracy of carbonation depth measurements. It does not seek to define any particular exposure condition for determining carbonation resistance of concrete for specific applications, and so it is applicable for testing specimens that have been exposed to either natural or accelerated carbonation.

## Definitions

### Carbonation

The chemical reaction between carbon dioxide and the alkaline components (hydrous or anhydrous) within a cement paste. This process can lead to the formation of sparingly soluble carbonate minerals, and a reduction of the pH of the pore solution, as a function of time, exposure conditions, and the remaining uncarbonated cementitious phases.^1^

### Carbonation depth

The perpendicular distance from the sample surface to the mean position of the carbonation front. This is also called the depth of carbonation, and it is abbreviated here as *d*_k_.

### Carbonation front

The location in the sample at which an observable pH change occurs. Material between the sample surface and the carbonation front is considered *carbonated*; material deeper into the sample than the carbonation front is considered *uncarbonated*.^2^

The reduction of the pH value at the carbonation front can be made visible by the colour change of a suitable indicator. However, it is important to note that pH indicators solely reflect a reduction in pH and cannot confirm that this is caused exclusively by carbonation. When other potential causes of pH reduction are present (e.g. exposure to SO₂, acids, or pure water), the decrease in pH should not be attributed solely to carbonation.

## pH indicator solutions

For determining carbonation depth, several indicators can be used, including those listed below. Historical test methods, including RILEM CPC-18 (the forerunner of this Recommendation) were almost always based on the use of phenolphthalein as a pH indicator. However, phenolphthalein is listed in the European Chemicals Agency REACH Regulation, Annex 2, as a candidate for listing as a substance of very high concern,^3^ so there is strong interest in testing and validating alternative indicators.

- **Phenolphthalein solution**: A solution of phenolphthalein powder in ethanol-water or another suitable solvent can be used. A commonly used preparation involves 0.8 g of phenolphthalein dissolved in a mixture of 70 mL of deionised water and 30 mL of ethanol, but different existing national standards provide different concentrations of the indicator, and/or different water/ethanol ratios [3].

In concrete with supplementary cementitious materials (SCMs), it is not always possible to clearly identify colour change boundaries, when carbonation depths are determined using phenolphthalein solutions produced as recommended in standards (blends of water and an alcohol). In these cases, it is recommended to dissolve 1 g of phenolphthalein in 100 mL of ethanol instead.

Phenolphthalein turns from fuchsia at high pH, to colourless at lower pH, with a transition in the pH range 8.2–10.

*Warning: phenolphthalein is considered a hazardous substance due to its carcinogenicity, and its use requires appropriate safety provisions.*

- **Thymolphthalein solution**: Thymolphthalein, typically used in a solution at concentrations of between 0.1 and 1%, can be dissolved in ethanol or water or their blend.

Thymolphthalein turns from colourless to blue as the pH increases, specifically within the range of 9.3 to 10.5.

- **Curcumin solution**: A solution of curcumin in ethanol can also be used for determination of carbonation depth. Concentrations around 0.5% curcumin have been shown to provide valid visualisation of the carbonation front. Curcumin changes from red/brown at high pH, to yellow at lower pH, with a transition in the pH-range 7.4–8.6 [8].
- **Anthocyanin solution**: Naturally occurring anthocyanins, extracted from e.g. grapes, red cabbage, black carrot, and from petals of the Chinese violet cress, *Orychophragmus violaceus,* can be prepared as a solution in an ethanol–water mixture. The diversity in natural anthocyanins means that the observed colour changes, and transition pH values, vary between specific anthocyanins, and should be specifically tested and validated in more detail if they are to be placed into widespread use in the future [9, 10]. In the case of black carrot extract, colour transitions from reddish-purple to brown (pH 6–10) to dark yellow (pH 10–13) can be observed [11].

These alternatives to phenolphthalein are less hazardous and can also provide clear visual determination of pH changes. This makes them potentially preferable options for carbonation testing. However, further validation is required to determine the most appropriate solvents and concentrations for each, for standardisation purposes.

**Fig. 1:**
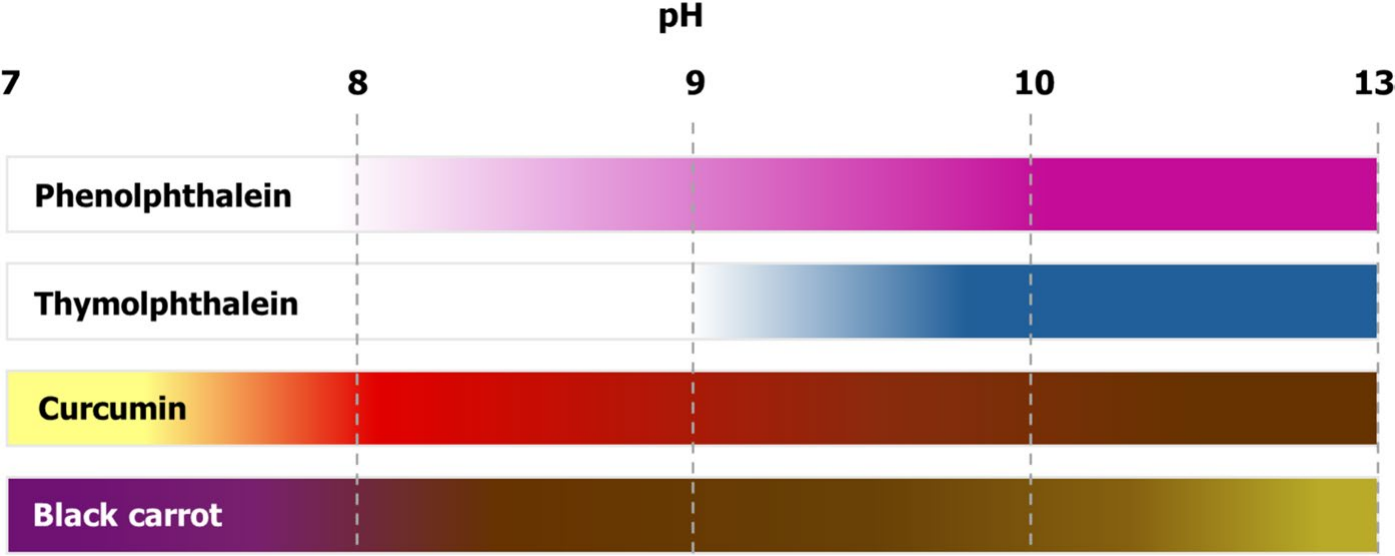
Colour changes of different pH indicators

It should also be noted that carbonation depth results obtained using different indicator types and/or solvents are not directly comparable, due to the differences in colour transition pH values, and potential mobilisation (leaching) of alkalis (Na^+^/K^+^) in the carbonated region depending on the quantity of water added along with the indicator as it is sprayed. Table 1 summarises the indicators for which positive experiences have been reported regarding the determination of carbonation depth. Colour changes identified using different pH indicators are shown in Fig. 1.

**Table 1:** Summary of potential pH indicator solutions for carbonation front detection

Indicator	Solvent composition (ethanol/water) (v/v)	Indicator concentration (wt.%)	pH transition range	Notes
Phenolphthalein	70/30	1	8.2–10.0	Safety precautions are required due to carcinogenicity
Thymolphthalein	90/10	0.1	9.3–10.5	Detects pH values closer to risk of rebar corrosion than phenolphthalein; Fades quickly; Safety precautions needed
Curcumin	100/0	0.1–0.3	7.5–9.2	Colour change less sharp; influenced by light and oxidation
Black Carrot	70/30	~ 2.5	6.5–8.0	Natural dye—considered a safe alternative

## Testing laboratory-prepared specimens

### Specimens: type, production, curing, and storage

For laboratory-prepared concrete specimens, prisms with a cross-section of 100 mm × 100 mm, that can be split by increments of approximately 50 mm at each testing interval, are suitable. In no case shall the shortest dimension of the prisms be less than 3 (ideally 5) times the maximum aggregate size. Cylinders may be used if carbonation is measured perpendicular to the flat, circular face (as is the case of cores extracted from structures in service). The use of carbonation depths measured along the radius should be limited to comparisons of such depths among specimens under identical exposure conditions and duration. However, carbonation coefficients derived from such radial measurements cannot be obtained using the square-root-of-time law, which assumes planar geometry and unidirectional diffusion. Radial diffusion in cylinders requires numerical modelling of Fick’s law in cylindrical coordinates to determine a valid carbonation coefficient [12].

During specimen preparation, it is important to avoid unwanted effects on the concrete surface which could impair measurement. For example, the use of stripping agents shall be kept to a minimum. It is desirable to follow the recommendations of a standardised method for making, pre-conditioning (when applied), and curing specimens, as these strongly influence the carbonation progress as discussed in detail in [3]. The type of curing chosen (duration of storage in water or moist conditions, accelerated hardening) shall be precisely defined and followed during the tests and reported with the results (see Sect. 5.3).

Climatic conditions of storage (temperature, relative humidity, CO_2_ concentration or partial pressure, and other relevant environmental) shall be defined and reported with the results (see Sect. 5.3). Specimens may be stored indoors or outdoors. For indoor storage a CO_2_ concentration of approximately 0.04%,^4^ a temperature of (20 ± 2) °C, and a relative humidity between 50 and 70%, controlled to within ± 5% of the nominal value, are recommended in general. However, environmental conditions as specified in the relevant national standards of the place of intended use of the concrete should be used, if these provisions exist locally. Standards such as EN 12390–10 provide detailed information about the control of storage conditions, and the design and implementation of appropriate storage environments. The history of CO_2_ concentration, temperature, and relative humidity in the storage environment shall be reported with the results (see Sect. 5.3).

For outdoor exposure testing, storage under cover (protected against rain) or without cover must be differentiated. Air must be able to always reach the test surfaces unhindered. For this reason, it is recommended to leave a free space of at least 20 mm around each specimen evaluated. Exposure to direct sunshine is likely to affect the temperature of the specimens and it is not desirable. Any accumulated snowfall shall be removed promptly to avoid blocking of the sample surfaces, and it is desirable to minimise damage to sample surfaces via exposure to freeze–thaw action.

The CO_2_ concentration, temperature, and relative humidity in the outdoor storage environment shall be monitored on a regular basis, preferably continuously using a digital weather-station or equivalent instrumentation, and be reported with the results (see Sect. 5.3).

### Testing

#### Exposure duration

For natural carbonation exposure, the following exposure durations are recommended for measuring carbonation depths: 0, 28, 56, 90 140, 180 days; 1, 2, (4, 8, 16…) years, after the first exposure to CO_2_. For accelerated carbonation testing, it is recommended to collect measurements after 0, 7, 14, 28, 56, 90, and 140 days of exposure, or until the specimens reach full carbonation. This is particularly important for concrete with reduced water/binder ratio and/or containing SCMs. The number of carbonation readings recommended here are based on the results of the interlaboratory testing conducted by the RILEM TC 281-CCC [15], where it was identified for concrete with SCMs that the carbonation rate generally stabilises after 90 days of exposure. Consequently, the carbonation coefficients determined at exposure durations beyond 90 days will provide information that is more representative of the material during its service life, than those identified at shorter exposure durations.

Testing before CO_2_ exposure (at 0 days) is required to determine to which extent carbonation has occurred during curing and preconditioning of the specimens; this may have a significant impact on the calculation of the rate of carbonation, particularly for materials that carbonate relatively rapidly and so are more prone to showing a non-zero initial reading [13, 14]. The longer testing periods (min. 1 year) should be chosen when a slow rate of carbonation is expected (e.g., storage outdoors without cover, or concrete with a particularly low rate of carbonation). In the case of a more specialised investigation, additional dates may be necessary. For concretes with SCMs, it is recommended to conduct measurements for a minimum of one year (with a minimum of 3–5 measurements) for calculating carbonation coefficients [15].

##### Determination of depth of carbonation

To measure the depth of carbonation, a slice of approximately 50 mm thickness shall be split or broken off one end of the prism, for each test interval. It is advised to avoid the use of cut or drilled surfaces for carbonation measurement, because the exposed unreacted cement grains can introduce errors into the measurement (see Sect. 6.2 for details on testing drilled cores). The slice shall be sufficiently thick to avoid any chance that carbon dioxide has penetrated from the end surface of the specimen to affect the observed measurements of the lateral surfaces. It may be beneficial to seal the end surface of the prism by applying a coating with an impermeable agent after each slice is split, to prevent this end-effect on carbonation measurements.

The depth of carbonation should be measured on the freshly broken surface. First, clear the broken surface immediately of dust and loose particles after breaking (without using water or causing abrasion), and then spray with the indicator solution without delay. Ensure that the indicator solution does not run off the surface; i.e,. do not use an excess of solution. If only a weak colouration, or none at all, appears on the treated surface, repeat the spraying after 30 min or when the surface has dried out. To temporarily stabilise the colouration, a resin may be sprayed over the surface after drying.

The time between spraying the indicator solution and the measurement of carbonation depth can influence the obtained carbonation depth values [3], and this effect may be different for specific binder types. For example, alkali-activated materials may in some cases require immediate readings, or readings within 15 min [14]. When using a phenolphthalein indicator solution, it is recommended that the measurements are carried out as soon as the surface has dried out and completed without a pause. If time and resources permit, it is recommended to conduct additional measurements at 15 min and (75 ± 10) min after the first spraying, to determine whether the time after spraying is a significant cause of uncertainty in the measurements for the specific materials tested. If the readings cannot be carried out within this period, it is recommended to use a fixing solution to retain the colour without change. Where a fixing solution has been used, the timing of the depth measurements is not critical. In all cases, the time between spraying the phenolphthalein solution and taking the readings of the carbonation depths must be reported.

For non-Portland cement concretes, identification of the optimal time for taking the readings might require preliminary testing. Some slag-rich cements show a very dark internal blue/green colouration that fades to white due to an oxidation reaction with air (which contains O_2_ and CO_2_) diffusing into the sample [16]. This dark colour may hinder the reading of indicator colour changes. It is possible that the carbonation and oxidation fronts within a slag-cement concrete sample will coincide, but this cannot be assumed to be the case.

Carbonation depth is to be measured with a calliper, as the distance between a point on a broken surface where the colour change occurs and the nearest edge of the surface, perpendicular to this edge. The precision of the measurement shall be to the nearest 0.2 mm. This level of precision is particularly important for short exposure periods, where differences of a fraction of millimetre can noticeably affect the calculated carbonation coefficients. Carbonation depths less than 0.2 mm are not differentiated. In practice, measurements using rulers or a folding ruler are possible; however, it should be noted that the precision in this case is significantly lower than when using a calliper. If this is the only available option, individual values shall be recorded to the nearest 0.5 mm, and average carbonation depths shall be reported to the nearest 0.2 mm, as indicated in Sect. 5.3.

When making manual measurements, preferably five, and no fewer than three, carbonation depth measurements per edge of a broken surface shall be made. The locations for the measurements shall be approximately equally spaced along the edge, and they must exclude the corner areas of the surface, where carbon dioxide has penetrated two sides at once. The carbonation depth of each side shall be calculated as the arithmetic mean of the measurements on that side, considering deviations as described below. The carbonation depth of the sample (*d*_k_) shall be calculated as the arithmetic mean of the measurements on all four sides.^5^

**Fig. 2:**
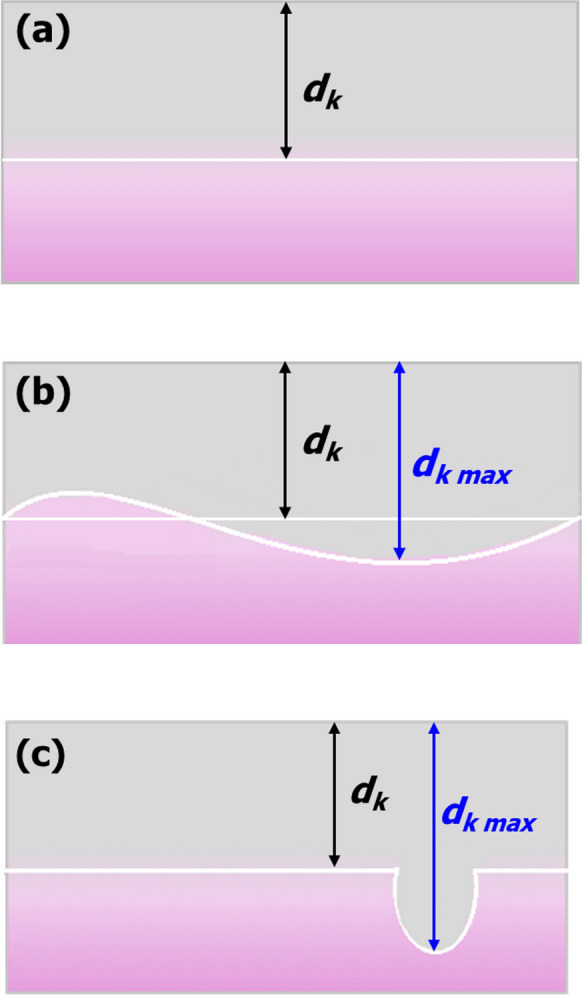
Illustrations of potential shapes of the carbonation front

When the carbonation front runs as a straight line parallel to the surface, the depth of carbonation *d*_k_ is determined as shown in Fig. 2a.

When the carbonation front is not homogeneous or cannot be clearly identified as changes of colour are detected not only parallel to the surface of the sample but also in preferential locations, as shown in Fig. 2b, the arithmetic mean of the individual readings of the carbonation depth as well as the maximum carbonation depth (*d*_k,max_) shall be reported. In such cases, it is recommended to record a photograph of the broken surface, analyse it via an image processing package as discussed below, and report carbonated areas instead. Also in this case, the mean as well as the maximum carbonation depth calculated by image analysis shall be reported, and the method adopted to calculate the mean shall be specified.

When the carbonation front runs parallel to the surface with isolated deeper carbonated areas (e.g. around cracks), as in Fig. 2c, the carbonation depths in such isolated areas are to be excluded from the calculation of the arithmetic mean of the carbonation depth, if the difference between the carbonation depth in the isolated area and the mean carbonation depth is 4 mm or more for concretes. If the difference is less than 4 mm for concretes, the carbonation depths in such isolated areas are included in the calculation of the arithmetic mean of the carbonation depths, as described in the previous paragraph on wavy carbonation fronts. In all cases, the arithmetic mean of the individual readings of the carbonation depth as well as the maximum carbonation depth shall be reported, and it is recommended to record a photograph of the broken surface.

In the case of concrete produced with large-sized natural aggregates, care must be taken to measure and report carbonation only in the hardened cement paste. In the case of concrete produced with large-sized recycled concrete aggregates (RCA), which might present a carbonated layer (due to weathering or carbonation pre-treatment), care must be taken to measure and report the carbonation only in the new hardened pastes. When a measurement point coincides with an aggregate grain surface, a line should be drawn connecting the carbonation front points on either side of the aggregate grain, and the measurement shall be taken as the distance from the specimen surface to such a line.

For more accurate measurements, high-resolution digital photographs of the pH indicator-sprayed specimens can be collected and used in image analysis methods to distinguish between carbonated and non-carbonated regions of the tested concrete. Image analysis methods may involve simple techniques, such as thresholding [17], and more refined approaches such as methods based on machine learning or other algorithms. Automated measurement systems offer higher accuracy and consistency in determining carbonation depth compared to manual readings. By using image analysis techniques or sensor-based detection, automated systems can precisely identify the boundary between carbonated and non-carbonated areas, reducing subjectivity and human error. However, unusual or inconsistent results should always be manually reviewed to ensure accuracy and account for factors such as irregular surface textures, variations in indicator application, or unexpected chemical interactions. A combination of automated measurement and manual verification provides the highest reliability, ensuring both precision and contextual assessment of carbonation depth.

### Test results and test report

All the measurements shall be expressed to the nearest 0.2 mm. The mean depth of carbonation shall be reported for each specimen. For laboratory specimens, this includes values from all sides. If the depth of carbonation of the floated or trowelled surface deviates considerably from those on the other surfaces, this shall be noted.

The test report shall include:

- General data:
  (i) Concrete mix design;
  (ii) Curing type (including relative humidity, temperature and duration);
  (iii) Climatic conditions (concentration of CO_2_, relative humidity, temperature; wind speed, orientation and precipitation during storage outdoors)
- Test data
  (i) Age of concrete;
  (ii) Form and size of concrete surface tested;
  (iii) Measuring apparatus/approach (in the case of image processing);
  (iv) Indicator solution details including indicator type and source, concentration, solvent used;
  (v) Time between spraying with indicator solution and measuring changes in pH;
  (vi) Mean depth of carbonation *d*_k_ to the nearest 0.2 mm, with details of how the carbonation front runs—if according to Fig. 2a: “regularly”, report *d*_k_; Fig. 2b: “irregularly”, report *d*_k_ and *d*_k,max_; Fig. 2c “regularly", report *d*_k_ and *d*_k,max_*;*
  (vii) If this is considerably different, record the depth of carbonation on the floated or trowelled surface;
  (viii) All results must include values of the standard deviation of mean values of all the measurements conducted per concrete mix design and exposure time.

## Testing samples obtained from structural concrete members

### Specimens

The determination of the depth of carbonation may be carried out using drilled cores taken from structures or prismatic specimens from other concrete items (e.g. slab or footpath) and subsequently split. The diameter of such a drilled core should be at least 50 mm, and in the case of prisms the shortest dimension must be no less than 3 times the maximum aggregate size.^6^

Concrete cores shall be taken and handled according to the standard provisions prevailing in the respective location, e.g., ASTM C42 [18], EN 12504-1 [19], or other comparable documents. Prism specimens need to be handled with similar care to cylinders.

### Testing

Determine the depth of carbonation in drilled cores or cut prisms immediately after drilling. If a longer storage period after drilling or cutting is unavoidable for any particular reason, the specimens shall be stored in CO_2_-free containers until they can be measured.

Measure the depth of carbonation, using the procedure of Sect. 5.2, applied to surfaces that are obtained by splitting the samples lengthwise, perpendicular to the surface of a structural member. Measurements carried out by applying the indicator solution directly to the outer surface of drilled cores are less accurate. However, when using cores extracted from a structure in service to assess multiple performance indicators (e.g., measuring also compressive strength in addition to the carbonation depth), testing the drilled surface for the carbonation depth may be justified to some extent by the need to maximise the use of each core and minimize destructive testing.

### Test results and test report

The depth of carbonation shall be reported to the nearest 0.2 mm. In reporting this measurement, disregard the thickness of any render or plaster that was applied to the concrete surface. The test report shall contain, in addition to the items given in Sect. 5.3, the following details:

(i) Identification of the structure;
(ii) Location of drilling and the orientation of the surface exposed to CO_2_ in the building (vertical or horizontal; geographic orientation);
(iii) Nature and thickness of any surface coating or treatment (e.g., render or plaster) that was present on the surface;
(iv) Date of drilling and of testing;
(v) General assessment of the concrete (structure of the concrete, aggregates, pores).

## Interpretation

The interpretation of the results of tests carried out according to this Recommendation depends on the actual goal of the carbonation depth measurement, and often needs specialised expertise in cement chemistry, concrete materials science, and corrosion. This will be illustrated below, examining the two probable case scenarios.

In many cases, carbonation depth measurements are carried out for assessing the risk for corrosion of (carbon) steel reinforcement, such as in condition assessment of reinforced or pre-stressed/post-tensioned concrete structures. While a decrease in pH is indeed generally associated with an increased risk for corrosion of carbon steel, because the steel passivity may be destabilised (locally or uniformly), there exists no clear and universally valid pH threshold below which corrosion occurs and above which corrosion does not occur. Various additional factors strongly influence whether steel depassivation may occur, and at what rate corrosion may proceed. These factors include the concentration of different substances in the concrete pore solution, including chloride, sulfate, and carbonate species, and dissolved oxygen. Moreover, the temperature, the moisture state of the concrete, and its pore structure, as well as the metallurgical and surface properties of the steel, are also decisive factors controlling the corrosion process. Thus, the pH of the concrete matrix surrounding the steel (and/or the carbonation depth) alone is not a reliable indicator to assess the corrosion risk of steel in concrete. Additional information on the conditions in the concrete, along with specialised expertise in corrosion and concrete materials science, is needed for a holistic assessment. Relying exclusively on pH values or carbonation depths may lead to significantly under- or overestimating the risk of steel corrosion in concrete.

If pH values or carbonation depths are measured in the context of carbon sequestration in concrete, e.g. to quantify the amount of CO_2_ sequestered, both the cementitious material under investigation and the pH indicator selected need careful consideration. This is because carbonation depth measurements determined by a pH indicator are neither an accurate nor a direct quantitative measurement of the type and amount of carbonates formed in the material. Consequently, detailed information on the various phases present in the cementitious matrix both before and after carbonation is required, combined with knowledge about the pH values, or pore solution chemistries in general, that are in equilibrium with these different phases. Such information may be obtained from mineralogical characterisation. Without such knowledge and deep understanding of the processes occurring at the fundamental level, quantifying the amount of sequestered CO_2_ is not possible, because information is required regarding the actual extent of the conversion of cementitious phases to carbonate minerals that correspond to the conditions within and outside the carbonation layer. The assumption of a sharp (zero-thickness) carbonation front, with uniformly carbonated material outside and uniformly uncarbonated material inside, is not sufficiently accurate to give high-quality results in calculations of CO_2_ uptake in this context.

## Footnotes

1 This Recommendation specifically addresses pH reduction as a method by which carbonation may be identified. Other methodologies are possible, including direct determination of the formation of carbonate phases within the cementitious microstructure by various spatially-resolved analytical methods. These may give similar or differing measurements of the carbonation depth depending on the nature of both the sample and the testing methodology. For the purpose of this Recommendation, the carbonation depth is defined according to the pH change.

2 This definition is based on the simplification of a sharp carbonation front, clearly delineating carbonated from uncarbonated regions. In practice, the carbonation front will be a region of finite thickness, but the single-point determinations based on the use of a pH indicator do not in general enable a non-zero carbonation front thickness to be identified, and so it is treated as a single point for the purposes of this Recommendation.

3 Full information is provided at https://echa.europa.eu/candidate-list-table/-/dislist/details/0b0236e1807dc152

4 Accelerated tests of shorter duration may be carried out using higher CO_2_ concentrations. At present no universal agreement exists as to the value of short tests using considerably higher CO_2_ concentrations. Particularly for cements with low Portland clinker (or CaO) fraction, there are indications that high CO_2_ concentrations lead to carbonation rate measurements that require correction factors or do not accurately represent performance under natural exposure conditions.

5 This averaging procedure enables accounting for scenarios where unequal numbers of measurements are taken per side of the sample.

6 If only an estimate is required, e.g., the purpose of the investigation is only to gain information regarding whether the carbonation front has already reached reinforcing steel, then the concrete surface may be broken by chisel and the test carried out directly on site.
